# Multi-Network Co-expression Analysis Enhances Biological Insights from Single-Cell Gene Expression

**DOI:** 10.1007/s12539-025-00806-3

**Published:** 2026-02-06

**Authors:** Alicia Gómez-Pascual, Araks Martirosyan, Katja Hebestreit, Andrew Kottick, Michelle Mighdoll, Victor Hanson-Smith, José Luis Mellina-Andreu, Alejandro Cisterna, Matthew G. Holt, Grant Belgard, Sebastian Guelfi, Juan A. Botía

**Affiliations:** 1 Information and Communications Engineering Department, University of Murcia, 30100 Murcia, Spain; 2 VIB Center for Brain & Disease Research, KU Leuven, 30001 Leuven, Belgium; 3 Verge Genomics, South San Francisco, 94080 USA; 4 The Bioinformatics CRO, Orlando, 32771 USA; 5 Department of Neurodegenerative Disease, UCL Queen Square Institute of Neurology, University College London, WC1N 3BG London, United Kingdom

**Keywords:** Dopaminergic neuron, Gene co-expression network, Multiple models, Parkinson’s disease, Single-cell RNA sequencing, Sparsity

## Abstract

With the advent of single-cell and single-nucleus RNA sequencing (sc/snRNA-seq), we can build cell-type-specific gene co-expression networks (GCNs). However, the high sparsity of scRNA-seq data limits GCN construction. We present scCoExpNets, a framework to create and annotate single-cell GCNs. For each cell-type cluster, scCoExpNets generates an initial GCN (T_0_) from its expression matrix. To reduce sparsity, pseudo-cells are created and used to build an alternative GCN (T_1_). This process is repeated through several iterations, thus creating multiple matrices and multiple GCNs for the same cell type. scCoExpNets was applied to an snRNA-seq dataset from the substantia nigra pars compacta (SNpc) from post-mortem samples of 13 controls and 14 Parkinson’s disease (PD) cases. Thanks to the creation of multiple GCNs for each cell type cluster, scCoExpNets detected that 95.84% of the T_0_ modules change their gene composition after sparsity reduction. In consequence, new biological annotations emerge, including 183 T_*i*_ GO subgraphs not detected at previous iterations, 159 GO subgraphs that expanded, and 157 GO subgraphs that specialized T_0_ GO subgraphs. Finally, we showcase the capabilities of scCoExpNets to detect a well-maintained dopaminergic module enriched with ferroptosis suppressors (*P*_adj_<0.004), which was replicated in two independent SNpc datasets (3.39 and 12.17 *Z*-summary score). The scCoExpNets R package and the GCNs created for all cell types are available on GitHub.

**Graphical Abstract:**

**Figure:**
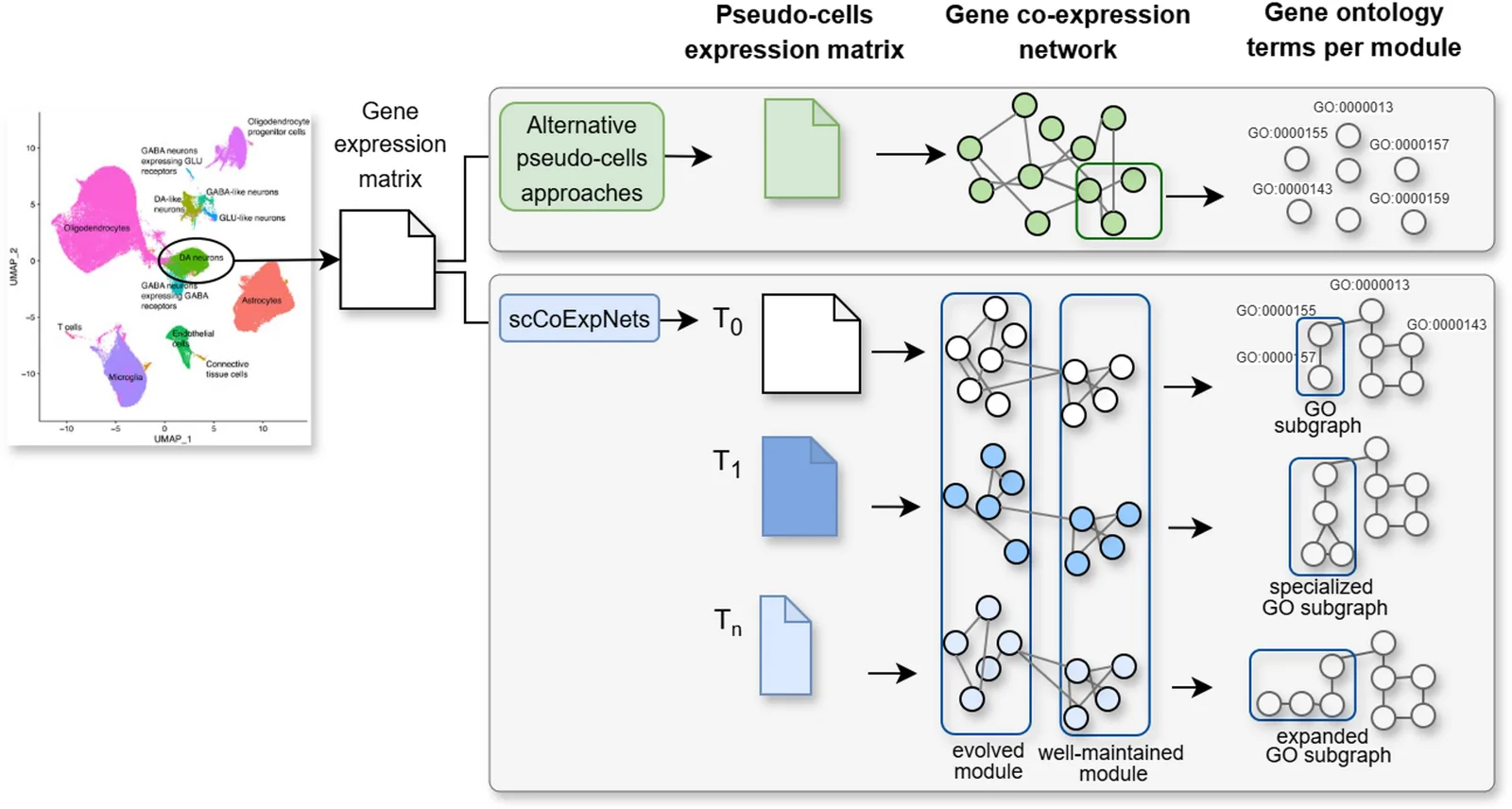

**Supplementary Information:**

The online version contains supplementary material available at https://doi.org/10.1007/s12539-025-00806-3.

## Introduction

Gene co-expression networks (GCNs) have been widely used for the discovery of novel pathways and gene targets in complex human diseases [1–5]. GCNs consist of nodes, which represent genes, and edges that indicate the association between genes. The connection strength between two genes is usually measured as the correlation between their gene expressions using the absolute correlation (i.e., unsigned) or keeping the sign of the correlation (i.e., signed). To determine if two genes are really connected, a hard threshold can be used, i.e., two genes are linked if the correlation between their expression profiles is over a threshold. However, it is not very meaningful to represent connections as binary information from a biological view; instead, weighted connections are the most common approach [6]. Once all gene connections are measured, unsupervised clustering is applied to group genes with similar expression patterns into modules. Finally, the most relevant modules are selected based on their association with donor traits (i.e., diagnosis or age) and/or gene information (i.e., functional enrichment analysis). The interesting modules detected allow us to discover new pathways and gene targets in specific scenarios. GCNs are commonly created from both microarray and bulk RNA sequencing data (bulk RNA-seq). However, bulk RNA-seq data do not provide the granularity to study the role of each cell type in both health and disease. Although it is possible to identify bulk RNA-seq modules enriched for specific cell type markers, it does not necessarily mean that these signals point to the most expressed genes, but to those from the most abundant cells.

Fortunately, single-cell RNA sequencing (scRNA-seq) offers the granularity to discover gene pathways involved in each cell type. scRNA-seq data is characterized by high sparsity because of low amounts of mRNA in individual cells and/or inefficient mRNA capture [7]. Therefore, the conventional Pearson and Spearman correlation coefficients are often not reliable for scRNA-seq data, especially for genes whose expression values are highly sparse. To address scRNA-seq sparsity, new methods have emerged, including: co-expression tables analysis (COTAN) [8], which focuses on zeros and their joint distribution to directly infer gene relations; scLink, which uses a filtering process to select only accurately measured read counts [9]; and single-cell gene co-expression network analysis (scGENA) [10], which applies an imputation method to estimate expression levels by borrowing information across genes. However, most of the new methods developed to address single-cell sparsity rely on the concept of *metacells*, defined as a group of scRNA-seq cell profiles that are statistically equivalent to samples derived from the same RNA pool. Based on this concept, Baran et al. [11] developed MetaCell, a new method for inferring and using metacells. A few years later, they improved their algorithm and created Metacell-2 [12]. Other methods based on this same concept include SuperCell [13], single-cell aggregation of cell states (SEACells) [14], and high-dimensional weighted gene co-expression analysis (hdWGCNA) [15]. To use any of these methods, developers propose some recommendations about the number of metacells to create (e.g., one metacell for every 75 single-cells). However, developers also pointed out that the optimal number of metacells is largely dependent on the biological structure of the data [14]. Consequently, it is not an easy task for the user to find the optimal number of metacells for a specific dataset. Additionally, these methods do not provide an effortless way to compare GCNs created from different pseudo-cell sizes. This would be beneficial since there is no established sparsity value that we can rely on when interpreting the calculated correlations.

To address this limitation, we propose scCoExpNets (single-cell multiple network co-expression analysis) a new easy-to-use multi-model method to address single-cell sparsity based on the concept of *pseudo-cells*. It is an easy-to-use method because the user does not have to decide the optimal number of pseudo-cells to create. Instead, for each cell type, scCoExpNets creates a set of expression matrices with successively decreasing sparsity ratios, and each matrix is used afterwards to create its corresponding GCN. It is a multi-model method because the GCNs created are interlinked and complementary models of the cells under study. They are interlinked because they share common insights on the transcriptomics of those cells, and they are complementary because each model contributes to the overall picture with new insights. Having multiple models helps reinforce the reliability of common insights, and, at the same time, alternative signals can be extracted from transcriptomic data. Managing multiple models is also made easy by the software by using new ways to annotate the modules, new ways of GCNs visualizations, and automatic detection of reliable and potentially interesting modules. We applied scCoExpNets on a single-nucleus RNA-seq dataset (snRNA-seq) from human post-mortem substantia nigra pars compacta (SNpc) tissue of 13 controls and 14 Parkinson’s disease (PD) cases. From these, we generated a set of GCNs for each cell type to characterize their role in both healthy conditions and PD pathology. We focus our attention on the dopaminergic neuron (DN) GCNs since the degeneration of midbrain DNs within the SNpc is a pathological hallmark of PD [16].

## Materials and Methods

### Post-mortem SNpc Donors’ Selection

The discovery cohort consisted of post-mortem human SNpc tissues from 27 donors collected from the Oregon Brain Bank at Oregon Health and Science University (OHSU), a human tissue repository for brain research studies. It included a total of 13 controls (eight males and five females) with a mean age of 75 years and 14 cases diagnosed with PD (10 males and four females) with a mean age of 82 years (see Supplementary Table S1). The average RNA integrity number (RIN) was 7.15, and the average post-mortem interval (PMI) was 21.62 h. The diagnosis (PD or control) was confirmed by a neuropathologist at OHSU after histological examination of the brain tissue. Controls did not have any other observable neurological conditions at the time of death. All PD samples showed Lewy body presence in midbrain and limbic brain areas, while 44.4% also had it in the neocortical area (see Supplementary Table S1).

### Nuclei Isolation and Single-Nucleus RNA Sequencing

The tissue samples were processed at the Vlaams Instituut voor Biotechnologie, in Flanders, Belgium, where the sample libraries were prepared. 2 mm biopsy punches were collected from the SNpc, and nuclei were isolated and prepared as per [17]. The single-nucleus libraries were sequenced using Illumina HiSeq4000 10x Genomics Single Cell V2.

### Preprocessing of snRNA-seq Data

Raw sequencing data were preprocessed using 10x Genomics Cell Ranger 3.0.2 software [18]. For quantification of unspliced mRNA in the snRNA-seq dataset, a reference transcriptome was generated from the pre-built GRCh38 assembly provided by 10x Genomics, in which exonic and intronic regions of each transcript were merged into a single exon, as recommended for Cell Ranger v3.0.2. After generating single nuclei gene counts for each of the samples, we aggregated all samples with equalized read depth to avoid batch effects. Raw unique molecular identifier (UMI) counts were generated by the Cell Ranger software. Then, Seurat v3 R package [19] was used to remove the *MALAT1* gene, which is known to be very highly expressed in snRNA-seq data and drive the cell clustering. Then, degraded nuclei were filtered out, including: (1) nuclei with > 5% of UMI counts coming from mitochondrially encoded genes through the PercentageFeatureSet function, (2) nuclei with low RNA-content (< 500 transcripts), and (3) doublets having high RNA-content (> 6000 transcripts).

### Clustering and Cell Type Identification

Cell type identification was carried out using the Seurat v3 R package. To this end, the following steps were applied: (1) SCT normalization of each sample separately regressing out the percentage of mitochondrial genes with the SCTransform function; (2) integration of cells across samples using the SelectIntegrationFeatures (nfeatures = 6000), PrepSCTIntegration, RunPCA, FindIntegrationAnchors, Reduce and IntegrateData functions; (3) principal component analysis (PCA) on integrated data with the RunPCA function; (4) UMAP dimensionality reduction with the RunUMAP function (50 dimensions); (5) cell clustering based on PCs using the FindNeighbors (50 dimensions) and FindClusters function (resolution = 0.6). Finally, the differentially expressed genes (DEGs) of each cell type cluster were identified through the findMarkers function based on a Wilcoxon Rank Sum test and a logFC threshold of 0.5. *P*-value adjustment was performed using Bonferroni correction based on the total number of genes in the dataset. The markers found in combination with an internal catalog of brain cell type markers were used to assign cell types to single nuclei. The same procedure was applied to the discovery and the replication datasets separately. Supplementary Fig. S1a shows the UMAP projection of the cell type annotated for the discovery, while Supplementary Fig. S1b shows the average expression of key cell-specific markers used to name the cell types.

### scCoExpNets Workflow

The most popular method to create GCNs is the weighted gene co-expression network analysis (WGCNA) [20]. This method was initially developed to use bulk RNA-seq or microarray data. Here, we developed scCoExpNets, a new software based on WGCNA, but adapted to scRNA-seq data to create cell-type-specific GCNs. The scCoExpNets workflow for each cell type is based on the following steps: (1) reducing scRNA-seq sparsity through several iterations by creating pseudo-cells; (2) constructing the corresponding GCN from each gene expression matrix (original and pseudo-cell matrices); (3) annotating the module of each GCN separately; (4) detecting interesting modules through pseudo-cells iterations; (5) visualizing the interesting modules. That is, scCoExpNets provides innovation in almost all steps of the pipeline. First, scCoExpNets provides a new and simple way to create pseudo-cells and reduce sparsity. Additionally, it offers new ways of annotating the modules (in addition to the already known). Then, the software includes its own mechanism to automatically detect interesting modules based on customized user criteria. Finally, it creates diverse kinds of plots to represent the most relevant features of the modules detected. That is, only the functions to create a GCN remain intact since WGCNA is a popular and standardized method to create GCNs.

#### Pseudo-Cell Approach to Tackle Sparsity

**Fig. 1:**
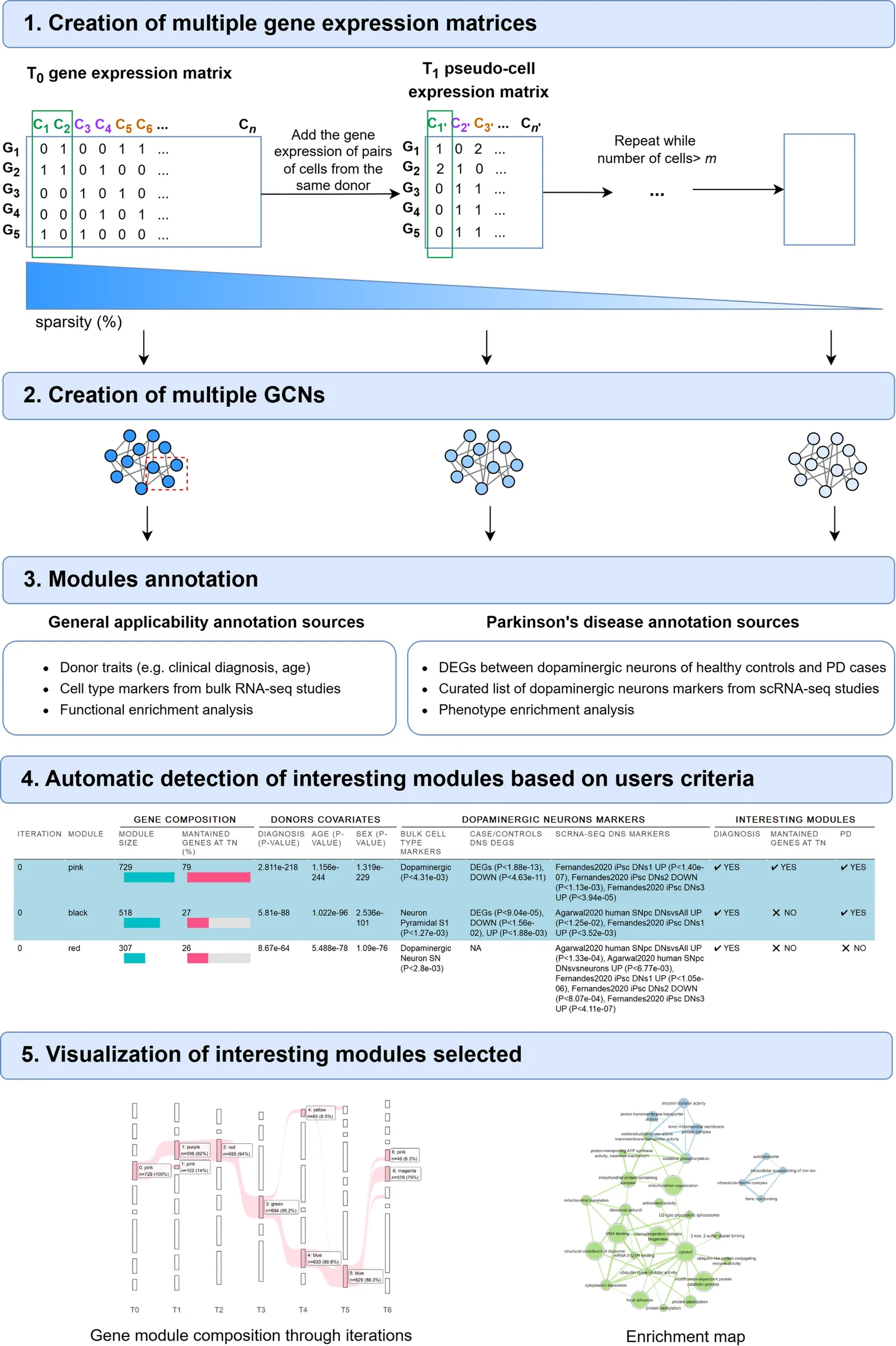
scCoExpNets workflow. Step 1: scCoExpNets addresses scRNA-seq data sparsity by creating pseudo-cells. To this end, gene expression of cell pairs from the same cell type and individual is added to create a pseudo-cell expression matrix, significantly less sparse and half the size than the predecessor. The process is repeated while the number of pseudo-cells is over *m*. **Step 2**: Each expression matrix is used to generate its corresponding GCN. **Step 3**: Modules in GCNs are first annotated using conventional sources of annotation, including the association of module eigengenes with donor traits (e.g., disease status or age), functional enrichment analysis, and cell type marker enrichment obtained from bulk RNA-seq studies. Additionally, scCoExpNets provides specific ways of annotating the modules, which include a list of curated DN markers obtained from scRNA-seq studies and phenotype annotations. **Step 4**: scCoExpNets automatically detects interesting modules based on users’ criteria. By default, interesting modules are defined as modules significantly correlated with donor diagnosis, and users can choose between well-maintained and evolved modules. **Step 5**: Once a module of interest is selected, scCoExpNets provides a Sankey plot to represent how genes are distributed through pseudo-cell iterations and an enrichment map to show the functional annotations connected to each other

scCoExpNets addresses scRNA-seq data sparsity using a new strategy to create pseudo-cells (Fig. 1, step 1). To this end, we define a pseudo-cell as the result of adding the gene expression of two cells that belong to the same donor and cell type. We start with the initial gene expression matrix of a cell type, and we use it to generate a GCN, denoted as T_0_. After that, scCoExpNets generates a pseudo-cell expression matrix using pairs of cells of the same donor from the previous iteration. This expression matrix is used to create the corresponding GCN, denoted as T_1_. The process of pseudo-cell creation is repeated while the newly created gene expression matrix T_*i*_ (*i* > 1) has at least *m* pseudo-cells in size (100 by default). Let *m* be the minimum pseudo-cells to create a GCN and *n* the number of T_0_ cells, and then scCoExpNets creates up to log_2_(*n/m*) GCNs for each cell type.

#### GCN Construction

The scCoExpNets algorithm works separately for each cell type. It starts with the original expression matrix and creates successive pseudo-cell matrices. scCoExpNets uses each matrix to create a GCN using WGCNA-based functions (Fig. 1, step 2). First, scCoExpNets corrects the gene expression matrix for confounding effects through the svaseq function from the sva R package [21]. In our case study, expression matrices were corrected for the technical effects of RIN and PMI, but not for the biological effects of age or sex since they are considered risk factors of PD [22, 23]. Then, a GCN is created from the preprocessed expression matrix using the getDownstreamNetwork function from the CoExpNets R package [24]. Then, the method generates the module membership (MM) of each gene in each module, which measures the relevance of the genes within the module and, therefore, it can be used to extract the most relevant genes (i.e., hub genes) of each module.

#### Gene Co-expression Module Annotation

Once the GCN modules are created, scCoExpNets annotates them using (1) conventional ways of annotating, such as module-trait correlations and functional enrichment analysis; (2) new ways of annotating more specific to the object of the study (Fig. 1, step 3).

##### Conventional Ways of Annotating the Modules

First, for each module within a GCN, scCoExpNets checks if the module eigengenes (i.e., the first principal component of the expression profiles of all genes in a module) are significantly associated with the donors’ traits based on a linear regression model approach. Traits (predictors) with significant *P*-values in the linear model are considered significantly associated with the eigengenes of the module. In addition, for each module of a GCN, a functional enrichment analysis is applied. To this end, we used the following sources: (1) Gene Ontology (GO) [25], which contains the subontologies of Cellular Component (CC), Molecular Function (MF) and Biological Process (BP), (2) Kyoto Encyclopedia of Genes and Genomes (KEGG) [26], and (3) Reactome pathway database (REAC) [27]. Functional enrichment analysis was applied through the gost function of the gProfiler2 R package [28]. Multiple testing correction was based on the g: SCS algorithm. Additionally, GO electronic annotations were removed. To compare the functional abundance between modules and GCNs, we defined the functional abundance statistic, which summarizes the significance level of the terms extracted from a functional enrichment analysis of a gene set. It is defined by

$$\:\sum\:_{i=1}^{n}\frac{-{\mathrm{l}\mathrm{g}}\left(P\left({t}_{i}\right)\right)}{n}$$

where *P*(*t*_*i*_) is the enrichment *P*-value for term *t*_*i*_, and *n* is the number of significant terms.

##### New and Specific Ways of Annotating the Modules

scCoExpNets also annotates the modules with phenotype-related terms obtained from the Human Phenotype Ontology (HPO) [29], CRISPRBrain [30], the Mouse Genome Database (MGD) [31], the Clinical Genome Resource (ClinGen) [32], the Genomics England PanelApp [33], the Comparative Toxicogenomics Database (CTD) [34], Orphanet [35], PsyGeNET [36], the Cancer Genome Interpreter (CGI) [37], and UniProt [38] databases. Phenotype-related terms from these databases were obtained through the PhenoEnrichGenes function of the PhenoExam R package [39]. Multiple testing was corrected via false discovery rate (FDR) estimation [40].

To select the most interesting modules for an object of study, scCoExpNets includes the capability to assess module enrichment for gene sets curated by the user. To this end, it applies a gene set enrichment analysis (GSEA) through the fgsea function from the fgsea R package [41]. Given the important role of DNs in PD, all DN gene modules were tested for enrichment of high-confidence PD gene makers obtained from the latest PD GWAS [42] and [43] work. Furthermore, to identify DN-specific modules, we tested our modules with DN markers obtained from three different sources: (1) cell type markers from bulk RNA-seq studies collected in the CoExpNets R package; (2) DEGs between DNs of PD cases and controls detected in our discovery dataset with the FindMarkers function from the Seurat R package; (3) our own curated list of DN markers obtained from scRNA-seq studies made up of 38 marker gene sets with variable sizes (6-207) (see Supplementary Table S2) [44–59].

#### Detecting GO Subgraphs in GCN Modules

Given a gene set and a gene annotation ontology such as the GO, a conventional enrichment analysis would show a set of terms significantly enriched for the genes in the gene set. Normally, a set of terms corresponds to subgraphs in the ontology. That is, terms may not appear isolated but rather accompanied by very related terms compounding a graph, with terms as nodes and ontology relations as arcs. The scCoExpNets tool identifies these ontology subgraphs and tracks them across pseudo-cell iterations. For each GO domain (CC, BP, and MF), subgraphs were detected using a depth-first search algorithm in the whole GO hierarchy through the GODag function from the goatools library [60]. The *is-a* semantic relation between GO terms was used to compound the subgraphs. Only the GO subgraphs with at least three terms in size were considered for downstream analysis.

To identify the T_0_ GO subgraphs that are maintained in all pseudo-cell iterations, the GO term overlap between each subgraph detected at T_0_ and the GO subgraphs detected in T_1_ iterations and later were estimated with a Jaccard-like index, i.e., $\:J\left(A,B\right)=\frac{\left|AB\right|}{\left|A\right|}$, where *A* refers to the terms of the subgraph at T_0_ and *B* to the terms in the subgraph at later iterations. We say that a T_0_ GO subgraph is well-maintained if the minimum overlap obtained in each pseudo-cell iteration is no less than 0.7.

In order to identify GO subgraphs emerging from pseudo-cell iterations rather than T_0_, we combine the Jaccard-like index mentioned above with other metrics such as the information content (IC), defined as the negative logarithm of the probability of observing the GO term *t* at the annotation sets in our modules. Terms with higher IC tend to appear at deeper levels of the ontology graph. The IC of GO terms was obtained through the calcICs function from the GOSim R package [61]. Based on these three measures, we define three types of new insights as follows. We identify a GO subgraph at T_1_ as new when it shows no overlap with any T_0_ GO subgraph (Jaccard index = 0). We also say that a subgraph at T_1_ is a specialization of a subgraph in T_0_ when both share at least 70% of the GO terms, but the T_1_ GO subgraph presents a higher average IC. Additionally, we say that a GO subgraph in T_1_ expands to a subgraph at T_0_ when its size is bigger than that at T_0_, shares at least 70% of its GO terms, and presents a lower or similar average IC. Finally, the T_1_ GO subgraphs that share < 70% of their terms with all T_0_ GO subgraphs are defined as shrunk subgraphs.

#### Automatic Detection of Interesting Modules

scCoExpNets includes a mechanism to automatically detect the most interesting modules based on customized users’ criteria (Fig. 1, step 4). By default, modules are characterized based on three groups of criteria: (1) their gene-level composition through pseudo-cell iterations; (2) their correlation with donors’ traits (e.g., clinical diagnosis, age); (3) their enrichment with gene sets curated by the user. Regarding the first criteria, we defined three different kinds of T_0_ modules: (1) well-maintained modules, which maintained > 70% of their genes together in the last iteration; (2) partially maintained modules, which keep 20%-70% of their genes together in the last iteration; (3) evolved modules, where < 20% of their genes remain together in the last iteration. The second and third groups of criteria are further explained in the gene co-expression module annotation section. Based on these groups of criteria, the user can define modules as interesting, for example, the well-maintained modules associated with the age of the donors and enriched with DN markers. To show the results, scCoExpNets generates an interactive table where the most interesting modules based on users’ criteria are highlighted.

#### Visualization of Interesting Modules

Once a module of interest is selected, scCoExpNets provides distinct types of visualizations to analyze the module in detail (Fig. 1, step 5). On the one hand, to evaluate how its gene composition is changing through pseudo-cell iterations, scCoExpNets generates a Sankey plot [62]. This plot is made up of as many columns as iterations (T_0_-T_*i*_), and each iteration has as many boxes as modules in the corresponding iteration. At T_0_, scCoExpNets highlights the module of interest and shows, using arcs, how these genes are distributed in the modules of different iterations.

Additionally, scCoExpNets creates an enrichment map to visualize and compare the GO annotations of a module across pseudo-cell iterations. It represents the results from pathway enrichment analysis as a network, where each node is a GO term, and edges represent the gene overlap between nodes. Related pathways are grouped into network clusters. This plot is created with the EnrichmentMap app (3.3.6) [63] from Cytoscape [64].

### Modules’ Replication in Independent Datasets

To assess whether the features of an interesting module are preserved in an alternative set of samples, two independent cohorts were selected. The first dataset consists of snRNA-seq data obtained from post-mortem human SNpc tissues dissected from seven samples provided by the Sepulveda brain bank. They were all male, three healthy controls with a mean age of 81 years and four PD donors with a mean age of 71 years. The average RIN was 7.73, and the average PMI was 12.06 h (see Supplementary Table S3). Additionally, the snRNA-seq dataset published by Wang et al. [65] was used as a second independent dataset. It is made up of 315,867 SNpc nuclei obtained from 32 donors (23 idiopathic PD cases and 9 controls, mean age 78 and 83 years, respectively). The two datasets were preprocessed using the same pipeline used for the discovery dataset (see Sects. 2.3 and 2.4). For the preservation test, from each cohort we selected the cluster with the highest expression of tyrosine hydroxylase, the main marker of DNs.

To evaluate whether the DN modules created with the discovery dataset replicated in the corresponding cluster of the two independent datasets, a preservation test was applied, which estimates the differences between what is observed and what is obtained by random permutation [66]. The preservation test is applied through the preservationOneWay function from the CoExpNets R package with 200 permutations, a maximum module size of 5000, and a maximum gold module size of 400. The preservation test generates a *Z*-summary score. Under the null hypothesis of no module preservation, *Z*-summary score < 2. *Z*-summary score > 5 (or 10) indicates moderate (strong) module preservation [66].

### scCoExpNets Applied in a Different Disease Context

To prove the robustness of our method and software, we evaluated scCoExpNets on an snRNA-seq dataset from a different disease context. To this end, the snRNA-seq dataset from Mathys et al. [17] was selected, which contains 80,660 single-nucleus transcriptomes from the prefrontal cortex of 48 individuals with varying degrees of Alzheimer’s disease (AD) pathology. Mathys et al. detected 44 cell type clusters in this dataset, grouped into eight major cell types, including astrocytes, microglia, and excitatory neurons. scCoExpNets was applied to each cell type cluster to create a set of GCNs for each one. The main statistics of these networks, including the number of modules, average module size, average number of functional enrichment terms per module, and average number of phenotype terms per module, were reported. Additionally, the dataset from Mathys et al. was also used to compare scCoExpNets performance with alternative pseudo-cell approaches (see Sect. 2.8).

### Comparison of scCoExpNets with Alternative Pseudo-cell Approaches

We assessed how effective scCoExpNets is when dealing with sparsity compared to alternative pseudo-cell approaches: SEACells, which provides exploratory capabilities, and hdWGCNA, which additionally creates GCNs. Both softwares rely on the notion of *metacell* but use different approaches to compute them. scCoExpNets was compared with these alternative pseudo-cell approaches in two different disease contexts: PD and AD. For the first one, we used the DN expression matrix of the discovery dataset (i.e., the Oregon dataset), since this is the main cell type affected by the disease. For the second one, the excitatory clusters 2 and 4 (Ex2 and Ex4) were selected from the dataset from Mathys et al. since they showed the highest correlation with high amyloid burden.

SEACells was applied to our unnormalized expression matrix by sequentially running the following functions with default parameters: pp.normalize_per_cell, pp.log1p, pp.highly_variable_genes, and tl.pca from the scanpy package [67]; core.SEACells (n_SEACells = 90), construct_kernel_matrix, initialize_archetypes, and fit from the SEACells package. Then, hdWGCNA was applied to our normalized expression matrix by sequentially running the following functions with default parameters: SetupForWGCNA, MetacellsByGroups (reduction = umap), NormalizeMetacells, SetDatExpr (assay = ‘SCT’), TestSoftPowers, and ConstructNetwork from the hdWGCNA R package.

To compare scCoExpNets DN pseudo-cell matrices used to generate T_0_-T_6_ with those from SEACells and hdWGCNA, we focused on sparsity and the number of pseudo-cells created. Then we compare modules created by scCoExpNets and hdWGCNA based on gene composition. To this end, we evaluated pairwise module overlap between the gene modules detected with scCoExpNets and hdWGCNA approaches using Fisher’s exact test through the overlapTable function of the WGCNA R package. Then, *P*-values were adjusted for multiple testing with FDR correction. Finally, functional abundance was estimated for each GCN.

## Results

### GCN Basic Statistics for Each Cell Type

In the discovery cohort, made up of 104,338 cells grouped into 24 different clusters, the expression matrices of the different cell types showed a high sparsity, with 77.2% (74.6%-79.9%) of zeros on average (Supplementary Table S4). This same trend was observed in the Sepulveda replication dataset, made up of 27,568 cells grouped into 20 annotated subclusters, which showed an average sparsity of 75.7% (71.8%-79.7%) across the different cell types (Supplementary Table S5). We addressed the sparsity problem of the discovery dataset using scCoExpNets. For each cell type, it collapses pairs of cells from the same individual to a new pseudo-cell expression matrix each time, using cells in T_0_ to create T_1_, pseudo-cells in T_1_ to create T_2,_ and so on. scCoExpNets reduced the sparsity ratio in each cell type expression matrix from T_*i*_ to T_*i*+1_ in 14.84% (13.77%-15.91%). Once all expression matrices were created for a specific cell type, scCoExpNets generated a GCN for each of them. At this point, we demonstrated that the order of the donors’ cells does not have an impact on the corresponding gene correlation matrix and, therefore, the resulting GCN. To this end, 100,000 different orders of DNs in the gene expression matrix were tested to create pseudo-cells. The resulting gene correlation matrix was quite similar between them (4.358 × 10^− 6^ mean Euclidean distance and 0.898 mean cosine).

On average, scCoExpNets created five GCNs for each cluster of the discovery dataset. The GCNs of all cell type clusters showed, on average, 20 (10–31) modules, with 523 (340–705) transcripts per module (Supplementary Fig. S2). The networks generated an average of 21 (15–27) functional terms per module from GO, KEGG, and REACTOME databases and 13 (9–17) phenotype-related terms per module (Supplementary Fig. S2). Interestingly, GCNs from DNs, the target of our study, showed the highest number of GO, KEGG, and REACTOME terms per module (39 terms on average) and the second highest number of phenotype-related terms per module (28 terms on average). The set of GCNs created for each cell type, as well as the corresponding functional enrichment, is available on GitHub.

Additionally, to demonstrate the robustness of our method and software on a different disease context, scCoExpNets software was applied to the snRNA-seq dataset from Mathys et al., which contains single-nucleus transcriptomes from the prefrontal cortex of 48 individuals with varying degrees of AD pathology. The gene expression matrix of each of the 44 cell type clusters showed high sparsity (81.26% on average). Using scCoExpNets, we managed to decrease the sparsity ratio by 13.81% on average after each pseudo-cell iteration. On average, scCoExpNets generated three GCNs for each cell type cluster, with a mean number of 30 modules (845 transcripts in size on average). Although these modules showed modest functional enrichment (eight enrichment terms amongst GO, KEGG, and REAC annotation databases on average per module corrected by module size), they stand out by a high number of phenotypic annotations (95 phenotype terms per module on average corrected by module size) (see Supplementary Fig. S3), which makes them highly interesting and useful.

### scCoExpNets Detects Well-maintained Modules and Evolved Modules Through Pseudo-cell Iterations

For each cell type, scCoExpNets creates multiple pseudo-cell expression matrices, thus multiple GCNs. This allows us to identify two types of modules: (1) those whose genes are maintained together through pseudo-cell iterations; (2) those whose genes are distributed in many different modules through pseudo-cell iterations, thus leading to new and potentially interesting modules. On average, only 4.16% of the T_0_ modules maintained 70% or more of their genes together in the last iteration across the different cell types (462 genes on average is the final size). Instead, 56.13% of the modules partially maintained their gene composition in the last iteration, i.e., between 20% and 70% of the original genes stuck together in the last iteration. The other 39.71% of modules maintained less than 20% of their original genes in the last iteration. These are considered evolved modules since they are transformed into something completely new after reducing sparsity. Supplementary Figure S4a shows the proportion of module types (maintained, partially maintained, or evolved) per cell type cluster. For all cell type clusters, we observed a higher proportion of partially maintained and evolved modules compared to well-maintained modules.

Although evolved modules can be seen as unreliable, later modules incorporating these genes and others showed at least similar or even higher functional abundance (see Sect. 2.5.3.1.) compared to the original module. Evolved modules at T_0_ showed a functional abundance of 1.80 (95% CI, 1.60-2.00). Through pseudo-cell iterations, these genes were rearranged in different modules, which showed an average functional abundance of 2.51 (95% CI, 2.36–2.66). That is, the difference of the functional abundance between T_0_ evolved modules and the modules created in later iterations with the same genes was − 0.71, therefore, the functional abundance was higher in the modules created in pseudo-cell iterations. A similar trend was observed with partially maintained modules. At T_0_, they showed a functional abundance of 3.07 (95% CI, 2.70–3.44) and modules at T_*i*_ created with these same genes reported a functional abundance of 3.34 (95% CI, 3.12–3.55), which leads to a difference of − 0.267. It suggests that modules at pseudo-cell iterations later than T_0_ tend to be more enriched in functional annotations justifying the use of a multi-model approach as we suggest.

In the same line, we have also demonstrated that partially maintained modules and evolved modules at T_i_ stand out by a high number of phenotypic annotations after FDR *P*-value adjustment. While maintained modules at T_*i*_ presented an average of 123 phenotype terms per module, partially maintained modules at T_*i*_ showed a higher number of phenotype-related terms with 147 phenotype-related terms per module on average. Finally, the evolved module at T_*i*_ also reported a high number of phenotype-related annotations with 190 terms per module. These results support the biological relevance of the modules created after pseudo-cell iterations.

### GCNs Created from Pseudo-Cell Matrices Provide New Insights

We wanted to go beyond functional abundance and look inside the actual knowledge we obtain from module annotation. Therefore, we focused on detecting GO subgraphs that are maintained through all pseudo-cell iterations, as well as new GO subgraphs that only appear when creating GCNs from pseudo-cell matrices, as well as those that specialize or complete previously existing GO subgraphs. Among all cell type clusters, scCoExpNets identified 586 T_0_ GO subgraphs (44.43% of all T_0_ GO subgraphs detected per module) that were well-maintained in all pseudo-cell iterations (four iterations on average) (see Supplementary Table S6). Within those, 49.15% (288 T_0_ GO subgraphs) belong to GO: CC (11 genes on average per subgraph), 33.45% (196 T_0_ GO subgraphs) to GO: BP (33 genes) and 17.41% (T_0_ 102 GO subgraphs) to GO: MF (13 genes). These T_0_ GO subgraphs maintained through pseudo-cell iterations are considered reliable GO subgraphs.

Thanks to the generation of multiple models across pseudo-cell iterations, scCoExpNets detected 112 new GO subgraphs (8.94% of all subgraphs in T_1_) with an average size of five terms (see Supplementary Table S7). They were also quite informative and specific, with an average IC of 7.89. Interestingly, among these new T_1_ GO subgraphs, we found a quite interesting subgraph related to the regulation of amyloid precursor protein formation and catabolic process (see subgraph 25, Supplementary Table S7). Additionally, we also detected 64 T_0_ GO subgraphs specialized at T_1_ (4.85% of the T_1_ subgraphs) (see Supplementary Table S8). Although T_1_ subgraphs showed a similar size compared to those at T_0_ (11 and 15 terms at T_1_ and T_0_, respectively), terms were slightly more specific and informative, with an average IC of 6, compared to the original T_0_ subgraphs, with an average IC of 3.76. Finally, we detected 71 GO subgraphs in T_1_ (5.38% of all the T_1_ subgraphs) that expanded others in the corresponding T_0_ subgraphs (see Supplementary Table S8). They showed a larger size compared with the corresponding T_0_ GO subgraphs (17 and 15 terms for T_0_ and T_1,_ respectively), but the terms were as specific, or less specific than those in the corresponding T_0_ GO subgraph (8.21 and 8.01 on average for T_0_ and T_1_, respectively).

Finally, to demonstrate that all pseudo-cell iterations—not just T_1_—provide new insights, we estimated the total number of specialized and expanded GO subgraphs that were detected at T_*i*_ but not in previous iterations. Specifically, we detected a total of 159 expanded GO subgraphs as well as 157 specialized GO subgraphs. In the same line, we also estimated the total number of new GO subgraphs detected at T_*i*_ but not in previous iterations, and we found a total of 183 completely new GO subgraphs. All the evidence points to the fact that creating GCNs from pseudo-cell expression matrices increases, specializes, and completes the biological insights of the GCNs from T_0_. Supplementary Figure S4b shows the number of maintained, shrunk, specialized, expanded, or completely new GO subgraphs detected at T_*i*_ but not in previous iterations for each cell type cluster. This figure shows that DNs, the focus of our study, are the cell type with the highest number of new insights, including expanded, specialized, and new GO subgraphs.

### scCoExpNets Selected Two PD-relevant Modules from DN GCNs

The DN GCN at T_0_ has nine gene modules with 432 transcripts per module on average. We showed that all these T_0_ DN modules were moderately preserved in the corresponding cluster of the snRNA-seq dataset from Wang et al. (23 idiopathic PD cases and nine controls), showing a 5.57 minimum *Z*-summary score (see Supplementary Fig. S5), suggesting a strong level of network replication in the algorithm. Then, we also tested whether the T_1_-T_*n*_ DN co-expression modules were also preserved in this independent dataset. We demonstrated that 100% (12 out of 12) of the T_*i*_ maintained modules (i.e., T_*i*_ modules that maintained > 70% of the genes of a T_0_ module) were well preserved in the independent cohort (9.28 min *Z*-summary score) (see Supplementary Fig. S5). On the other hand, 95.12% (39 out of 41) of T_*i*_ partially maintained modules (i.e., T_i_modules that maintained 20%-70% of the genes of a T_0_ module) were very well preserved in this new dataset (12.53 mean *Z*-summary score). Finally, only one DN module was classified as an evolved module at T_6,_ and this module was also strongly preserved in the independent cohort (19.19 *Z*-summary score). The preservation of almost all the modules (maintained, partially maintained, and evolved modules) in an independent cohort demonstrates the robustness of the modules created.

**Fig. 2:**
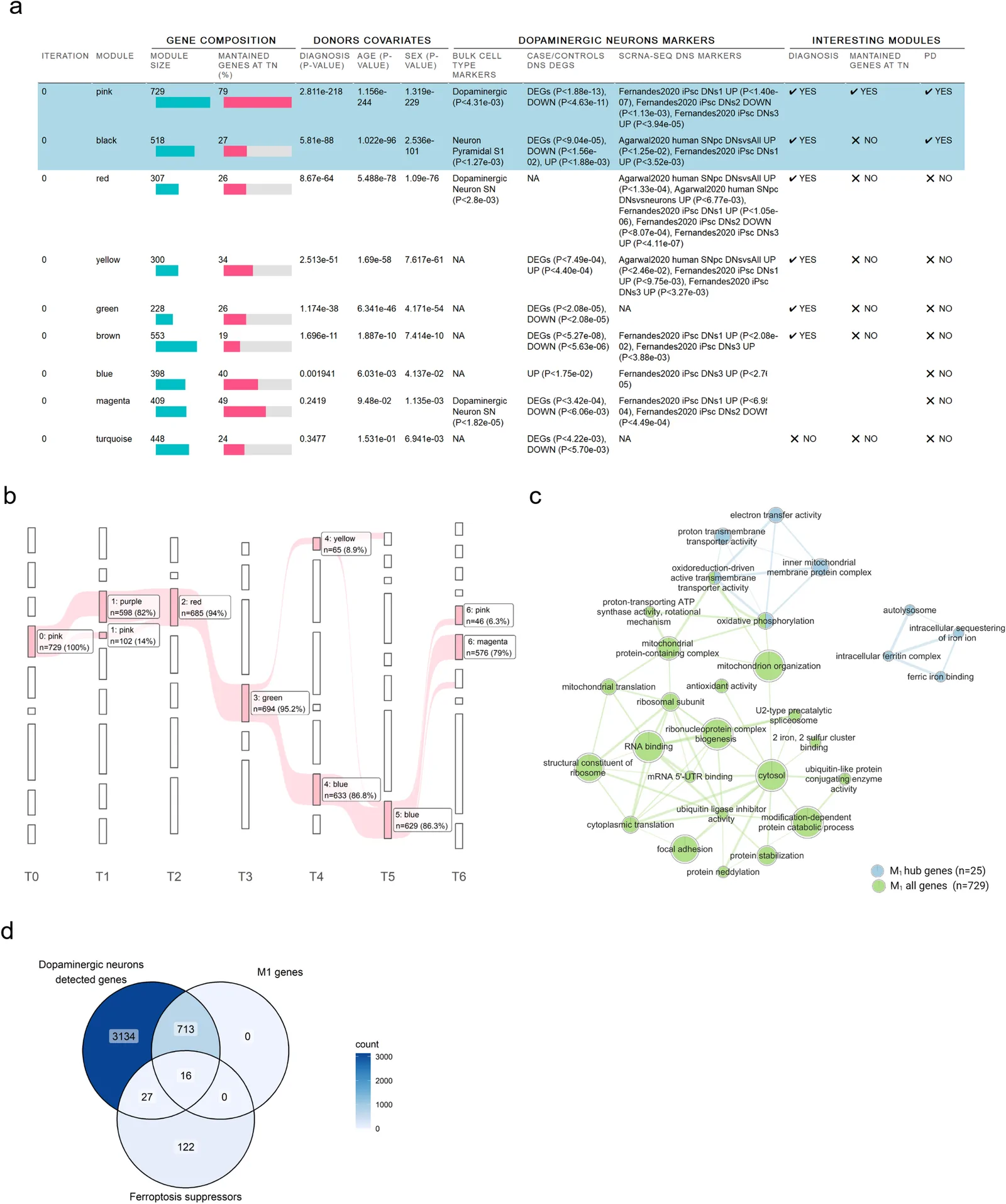
scCoExpNets selects PD-relevant modules from T_0_ DN GCN. **a** scCoExpNets facilitates module analysis of all GCNs created for a particular cell type by creating an interactive table that shows all relevant annotations (columns) for a set of modules (rows). scCoExpNets also detects the most relevant modules (highlighted in blue) based on users’ criteria. In this case, the table shows all relevant annotations for all T_0_ DN modules. The annotations represented in the table are grouped into four categories: (1) gene composition, which contains the module size and the percentage of genes that remained together in the last iteration; (2) donor covariates, which contains the *P*-value of the association between module eigengenes and donor covariates; (3) DN markers, which contain the *P*-value from the GSEA tested on case/control DN DEGs, where DN markers were obtained from both bulk and scRNA-seq studies; (4) interesting modules, which contain the criteria used to select the most relevant modules. In this case, we detected PD-relevant modules based on the following criteria: modules significantly correlated with clinical diagnosis, enriched with DN markers from both bulk and scRNA-seq studies and enriched with case/control DN DEGs. **b** scCoExpNets provides a Sankey plot to show how genes of a specific module are distributed in other modules through pseudo-cell iterations. The Sankey plot is made up of as many columns as pseudo-cell iterations and each column contains as many boxes as modules. In this case, the Sankey plot represents how M_1_ module genes from T_0_ DN GCN are distributed through pseudo-cell iterations. This module is made up of 729 genes at T_0_. In the first iteration (T_1_), 598 of those genes are maintained together in the same module while other 102 genes are also grouped together in another module. Finally, at T_6_, 576 genes are still together in the same module. **c** The enrichment map shows the most relevant GO annotations for the M_1_ module from T_0_ DN GCNs. Nodes represent highlighted driver GO terms. The size of the node is proportional to the number of M_1_ genes that take part in each term. The width of the edges is proportional to the number of genes shared between two nodes. Nodes represented in green are annotations exclusive to the whole module while nodes represented in blue are annotations exclusive to the top 25 hub genes. **d** Venn diagram shows the overlap between ferroptosis suppressors obtained from FerrDb V2 database and M_1_ genes from T_0_ DN GCN. We found 16 ferroptosis suppressors in the M_1_ module that were significantly represented in this module (GSEA *P*_adj_<0.004)

Once the robustness of the T_0_ and T_*i*_ modules was demonstrated, the criteria used to detect interesting modules in the context of PD were defined, which included: (1) modules showed a significant correlation with clinical diagnosis; (2) modules were enriched with DN DEGs between PD cases and controls; (3) modules were enriched with DN markers obtained from different sources (see Sect. 4.5.3.). Based on these criteria, scCoExpNets automatically selected M_1_ and M_2_ modules (see Fig. 2a). The main difference between the two modules selected is that M_1_ (pink in the table at Fig. 2a) was detected as a well-maintained module through pseudo-cell iterations (79% of its genes are still together in the last iteration) while M_2_ (black in the table at Fig. 2a) was classified as a partially maintained module, with 27% of their genes together in the last iteration. We decided to focus on the M_1_ module for downstream analysis since this module was preserved in the DN expression matrix from both the Sepulveda cohort (see Supplementary Table S9) and the dataset from Wang et al. (*Z*-summary score of 12.17, see Supplementary Fig. S5).

M_1_ showed a high correlation with donors’ clinical diagnosis (*P* < 2.811 × 10^− 218^), age (*P* < 1.156 × 10^− 244^), and sex (*P* < 1.319 × 10^− 229^). In addition, case/control DN DEGs were over-represented in this module (GSEA *P* < 1.88 × 10^− 13^), especially PD down-regulated genes (GSEA *P* < 4.63 × 10^− 11^). DN markers were also over-represented in this module, including (1) bulk DNs markers obtained from the CoExpNets R package (GSEA *P* < 4.31 × 10^− 3^) and (2) scRNA-seq DN markers obtained for different clusters of the Fernandes et al. study [56], including up-regulated genes from DNs_1_ and DNs_3_ clusters (GSEA *P* < 1.40 × 10^− 7^ and *P* < 3.94 × 10^− 5^, respectively), as well as down-regulated genes from DNs_2_ cluster (GSEA *P* < 1.13 × 10^− 3^). All this evidence suggests that M_1_ is a module highly specific to DNs. Furthermore, M_1_ generated an abundant annotation term set from both the functional and phenotype enrichment analyses. This includes 374 functional terms (21.97% of all terms in the network) and 91 phenotype terms (8.58% of all terms).

Additionally, M_1_ is stable in terms of transcript composition across successive pseudo-cell iterations (i.e., 79% of its genes are still together in the last pseudo-cell iteration) (see Fig. 2b). Accordingly, the functional abundance of this module is maintained throughout the iterations. M_1_ showed a functional abundance of 15.87 in T_0_ and 14.69 on average for the corresponding module in later iterations (the one with the highest proportion of M_1_ genes). Finally, we also found this module preserved in the DNs from the Sepulveda cohort (*Z*-summary score of 3.39). Moreover, the module is still preserved through pseudo-cell iterations, except for T_1_, whose *Z*-summary score is slightly under the cutoff (see Supplementary Table S9).

### PD-relevant DN Module and its Association with Ferroptosis

M_1_ module showed the biggest GO: CC subgraph maintained in all pseudo-cell iterations (40 terms in size). This subgraph was related to the ribosome, mitochondria, and extracellular vesicles (average IC of 6.26). Additionally, this module also showed the second biggest GO: BP subgraph (69 terms in size), associated with the electron transport chain (average IC of 5.05). In the same line, this module showed the second biggest GO: MF subgraph maintained in all iterations (15 terms in size). This subgraph was also associated with oxidoreductase activity and NADH dehydrogenase activity (average IC of 6.67). Although GO subgraphs detected at M_1_ were stable across pseudo-cell iterations, we wanted to have a more precise idea of possible function. To this end, we focused the analysis on the top 25 hub genes based on their module membership. Functional enrichment analysis showed that these hub genes were enriched for terms such as inner mitochondrial membrane protein complex (GO:0098800, *P* < 1.23 × 10^− 5^), oxidative phosphorylation (KEGG:00190, *P* < 6.13 × 10^− 6^), and ferritin complex (GO:0070288, *P* < 3.72 × 10^− 4^) (see Fig. 2c, Supplementary Table S10).

To gain more insight about the possible connection of M_1_ with iron metabolism and ferroptosis, we crossed its genes with a list of 152 ferroptosis drivers and 165 ferroptosis suppressors, all of them validated and tested in humans, obtained from the FerrDb v2 database [68]. Among 43 ferroptosis suppressors detected in our DN expression matrix, 16 belonged to M_1_ (GSEA *P*_adj_<0.004) (see Fig. 2d). Besides, M_1_ was the only module in the T_0_ DN GCN enriched with ferroptosis drivers or suppressors. Among the 16 ferroptosis suppressors detected, six of them were detected as highly representative genes in the M_1_ module since they showed a percentile in MM over 80%. These genes include ferritin heavy chain 1 (*FTH1*; 100% MM), glutathione peroxidase 4 (*GPX4*; 100% MM), brain expressed X-linked 1 (*BEX1*; 99% MM), ferritin light chain (*FTL*; 98% MM), iron-sulfur cluster assembly enzyme (*ISCU*; 86% MM) and serine and arginine rich splicing factor 9 (*SRSF9*; 80% MM). Among them, the following genes were down-regulated between PD cases and control DNs: *GPX4* (*P*_adj_ <4.65 × 10^− 13^, log_2_FC= −0.182), *BEX1* (*P*_adj_ <5.24 × 10^− 39^, log_2_FC = − 0.289), *ISCU* (*P*_adj_ <3.17 × 10^− 32^, log_2_FC = − 0.226), and *SRSF9* (*P*_adj_ <5.89 × 10^− 6^, log_2_FC= − 0.075). All this evidence may suggest that ferroptosis is disrupted in PD DNs.

### scCoExpNets Significantly Improves Biological Insights Produced by hdWGCNA

We compared scCoExpNets’s approach to deal with data sparsity with two alternative pseudo-cell approaches, SEACells and hdWGCNA, to demonstrate its suitability for GCN analysis. To this end, we focused on the DN expression dataset. The T_0_ DN expression matrix of the discovery dataset is characterized by 80.8% of zeros. SEACells reduced its sparsity up to 2.0% (90 pseudo-cells) and hdWGCNA up to 6.0% (404 pseudo-cells). scCoExpNets generated six pseudo-cell matrices with decreasing sparsity ratio, from 67.8% to 4.4%. The three methods get reasonably similar ratios (see Supplementary Fig. S6a). We also compared gene modules obtained with scCoExpNets and those obtained with hdWGCNA (SEACells does not create GCNs). First, a similar module size between approaches was observed, with a mean module size of 465 and 486 transcripts for scCoExpNets and hdWGCNA GCNs, respectively. In terms of module composition, all modules within all GCNs created by scCoExpNets were at least partially preserved in one hdWGCNA gene module after *P*-value adjustment (see Supplementary Table S11). This suggests that modules are preserved using an alternative pseudo-cell approach (see Supplementary Fig. S6b-h) in all network iterations.

In terms of functional abundance, the GCN generated with hdWGCNA reported a total of 52 GO subgraphs, of which 29 were maintained (55.77%), nine were expanded (17.31%), and eight were specialized (15.38%) in at least one of the scCoExpNets GCNs (T_0_-T_6_) (see Supplementary Table S12). Only six hdWGCNA modules (11.54%) were not maintained in any of the scCoExpNets GCNs. That is, 88.46% of the knowledge generated by the hdWGCNA GCN is collected by scCoExpNets GCNs, where 32.69% of its knowledge is improved (specialization or expansion). Additionally, the GCN created with hdWGCNA did not collect all the knowledge generated by scCoExpNets GCNs. Among the 70 GO subgraphs detected for T_0_ GCN of scCoExpNets, 40 of them (57.14%) were not found in the hdWGCNA GCN. After evaluating each of the scCoExpNets GCNs (T_0_-T_6_), we observed, on average, that 52.65% of the scCoExpNets GO subgraphs were not found in the hdWGCNA GCN.

To demonstrate the robustness of our method, we also compared the performance of the hdWGCNA and scCoExpNets methods using a completely different dataset. To this end, excitatory clusters 2 and 4 (Ex2 and Ex4) from the dataset from Mathys et al. were selected (see Sect. 2.7). We demonstrated that Ex2 GCNs generated with scCoExpNets gather most of the hdWGCNA GO subgraphs (88%), where 12% of the GO subgraphs are expanded, and another 12% are specialized. On the other hand, the hdWGCNA GCN does not collect most of the GO subgraphs of each scCoExpNets GCN (i.e., in each pseudo-cell iteration, an average of 66.73% of the scCoExpNets GO subgraphs were not found in the hdWGCNA GCN). Ex4 cluster followed a similar trend. scCoExpNets GCNs collected 72.1% of hdWGCNA GO subgraphs, where 6.98% of them were expanded and 4.65% were specialized. On the opposite side, hdWGCNA does not collect most of the GO subgraphs of scCoExpNets GCNs (an average of 71.73% of the scCoExpNets GO subgraphs were not collected by the hdWGCNA GCN). That is, for both clusters, Ex2 and Ex4, we demonstrated that the set of GCNs generated with scCoExpNets includes most of the hdWGCNA knowledge, while the hdWGCNA GCN fails short in gathering a considerable amount of all the annotations generated with scCoExpNets.

## Discussion

Here, we developed scCoExpNets, a new methodology that deals with scRNA-seq sparsity to create GCNs. For a specific cell type, scCoExpNets reduces the sparsity of its gene expression matrix by creating pseudo-cells, i.e., it collapses the gene expression of pairs of cells that belong to the same individual and cell type. Unlike alternative tools such as SEACells and hdWGCNA, which create a unique metacell expression matrix from the original expression matrix, scCoExpNets applies successive pseudo-cell iterations, leading to a set of gene expression matrices with a reducing sparsity ratio. Then, each gene expression matrix leads to a new GCN. That is, for each cell type, we create multiple GCNs, all of them seen as alternative and complementary views of the same phenomena. Our software includes all the functionalities needed to manage multiple models at the same time and detect the most interesting modules for our object of study. To this end, scCoExpNets provides new ways of annotating the modules, automatically detecting interesting modules, visualizing module composition, and analyzing GO subgraphs. Thanks to the creation of multiple GCNs for a specific cell type, scCoExpNets identifies gene co-expression modules whose gene composition is well-maintained through pseudo-cell iterations, which increases our trust in them (i.e., they are robust to sparsity) and also gene modules that evolve towards potentially more interesting gene groups in each iteration. In the same line, scCoExpNets detects T_0_ GO subgraphs that are well-maintained through pseudo-cell iterations, as well as T_*i*_ GO subgraphs that provide new insights not detected in previous iterations.

We showcase scCoExpNets on a snRNA-seq dataset of human post-mortem SNpc tissue of 13 controls and 14 PD cases to detect the most interesting DN modules since the degeneration of the DN in the brain is known to play a key role in the development of PD. scCoExpNets identified the M_1_ module from iteration T_0_ as one of the most interesting DN modules. The gene composition of this module was maintained through the pseudo-cell iterations, keeping 79% of its genes together in the last iteration. Additionally, this module was preserved in an independent cohort, which makes it more reliable. Functional enrichment analysis showed that the hub genes of this module participated in the regulation of iron metabolism. Moreover, this module was enriched with ferroptosis suppressors. All this evidence may suggest that ferroptosis is disrupted in PD DNs. In the same line, Do Van et al. [69] demonstrated that ferroptosis is an important cell death pathway for DNs in PD. In addition, treatments that protect against ferroptosis have shown therapeutic potential in PD [70, 71]. Although these findings demonstrate that the models of DNs created are coherent, as they validate previous findings about PD, new insights with potential applications in the context of PD have also been discovered. One of T_0_ DN modules was enriched with annotations related to Aggrephagy (REAC: R-HSA-9646399, *P* < 6.21 × 10^− 4^), defined as the selective degradation of protein aggregates by macroautophagy. Although modulation of aggrephagy could facilitate the clearance of pathological protein inclusions from neurons, the potential of this approach as a new treatment avenue in PD is still poorly explored [72]. Additionally, another T_0_ DN module was enriched with functional annotations related to L1CAM (i.e., L1CAM interactions, REAC: R-HSA-373760, *P* < 7.03 × 10^− 4^). This protein has been recently presented as a promising serum biomarker for individuals at risk of developing PD [73, 74]; however, this marker has been poorly studied at the DN’s level.

Finally, we compared scCoExpNets with alternative pseudo-cell approaches. Initially, we identified analogous alternatives for pseudo-cell generation; however, most of them were exclusively designed for visualization purposes, such as SEACells. Then, we compared scCoExpNets with hdWGCNA, one of the most popular tools to address scRNA-seq sparsity to create GCNs. Though all DN modules generated by scCoExpNets through iterations were at least partially preserved in one DN hdWGCNA gene module after *P*-value adjustment, significant differences emerged in the GO subgraph analysis. Specifically, 88.46% of the knowledge generated by the hdWGCNA GCN was collected by scCoExpNets GCNs, where 32.69% of the knowledge was specialized or expanded. In contrast, hdWGCNA only collected, on average, 47% of the scCoExpNets GO subgraphs. That is, scCoExpNets generates and extends more biological knowledge than hdWGCNA. In addition, scCoExpNets offers additional functionalities not available in hdWGCNA, which includes: (1) extensive module annotation; (2) generation and comparison of GO subgraphs; (3) quantification of module overlap through pseudo-cell iterations; (4) visualization of module composition progression through pseudo-cell iterations.

Our method has been designed to work on scRNA-seq data. However, there are ways in which it may be extended to leverage scenarios with different data modalities. For example, it may be extended to multi-omics building independent co-expression network families for each type of omics and correlate the models in a *post-hoc* analysis [75–77]. But this is applicable on the assumption that co-expression is a suitable modeling paradigm for all data types. This is usually not the case in regulatory networks when different data modalities imply different regulatory elements that operate differently but are expected to interact. As an example, the Single-cell Multi-omics Gene Co-Regulatory algorithm (SMGR) [78] integrates scRNA-seq and scATAC-sec through a regulatory network identifying regulatory signals linking gene expression and chromatin through ATAC-seq peaks.

## Supplementary Information

Below is the link to the electronic supplementary material.

Supplementary Material 1

Supplementary Material 2

Supplementary Material 3

Supplementary Material 4

Supplementary Material 5

Supplementary Material 6

Supplementary Material 7

## Data Availability

The discovery snRNA-seq dataset used in this research is available on Gene Expression Omnibus (GEO) under the accession number *GSE243639*. The raw SNpc snRNA-seq data from Wang et al. are available on GEO under the accession code GSE184950 and the prefrontal cortex snRNA-seq dataset from Mathys et al. is available on Synapse under the accession number syn18485175.

## Code Availability

The scCoExpNets R package is available on GitHub (https://github.com/aliciagp/scCoExpNets). Additionally, the set of GCNs created for each cell type of discovery snRNA-seq dataset and their functional annotations are also available in this repository.
