# Supplementary material for: Multi-Network Co-expression Analysis Enhances Biological Insights from Single-Cell Gene Expression
Source: Interdiscip Sci. 2026 Feb 6;18(4):1231–47. doi: 10.1007/s12539-025-00806-3 (PMC13694558; doi:10.1007/s12539-025-00806-3)
Supplement: Supplementary file 1 [file 12539_2025_806_MOESM1_ESM.docx]

**Supplementary Figure S1. The discovery substantia nigra pars compacta single-nucleus RNA-sequencing dataset. (A)** UMAP projection of cells annotated per cell type for the discovery dataset. **(B)** Average expression of key cell type markers across the cell types in the discovery dataset.

**Supplementary Figure S2. GCNs statistics per cell type from the discovery dataset.** Cell types are represented within each plot row. Y-axis of each plot refers to the iteration within which the GCN was created from T_0_ to T*_n_*, where the maximum number of iterations is seven. Plots at columns correspond to the features observed from networks including sparsity (%), number of modules, average module size, mean number of GO, KEGG and REAC terms per module and mean number of phenotype-related terms per module.

**Supplementary Figure S3. GCNs statistics per cell type of the snRNA-seq dataset from Mathys *et al.*** Cell types are represented within each plot row. Y-axis of each plot refers to the iteration within which the GCN was created from T_0_ to T*_n_,* where the maximum number of iterations is seven. Plots at columns correspond to the features observed from networks including sparsity (%), number of modules, average module size, mean number of GO, KEGG and REAC terms per module and mean number of phenotype-related terms per module.

**Supplementary Figure S4. scCoExpNets creates multiple GCNs for each cell type to extend the biological insights that can be extracted from scRNA-seq data. (A)** Proportion of modules’ types per cell type cluster. The X axis represents the cell type clusters detected in the discovery dataset (the number after the name indicates the cluster where each belongs since the same cell type can have different clusters assigned). The Y axis shows the proportion of T_0_ modules maintained, partially maintained and evolved through pseudo-cell iterations for each cell type cluster. Although it may appear that the proportions of modules are different between cell types, a common pattern is observed: most of the modules have significantly changed their transcript composition through pseudo-cell iterations, either partially or completely. That is, there is a low proportion of modules that are robust to sparsity and have maintained their transcript composition throughout the iterations. **(B)** Number of GO subgraphs detected per cell type cluster. The X axis represents the cell type clusters detected in the discovery dataset. The Y axis shows the number of: (1) T_0_ GO subgraphs maintained or shrunk at T*_i_* but not in previous iterations; (2) T_0_ GO subgraphs expanded or specialized in T*_i_* but not in previous iterations; (3) T*_i_* GO subgraphs that are completely new and did not appear in previous iterations. Thanks to the creation of multiple GCNs, scCoExpNets is able to detect T*_i_* GO subgraphs that provide new knowledge not detected in previous iterations. Dopaminergic neurons stands out as the cell type with the highest number of GO subgraphs that provides new insights.

**Supplementary Figure S5. Preservation of dopaminergic neurons co-expression modules in the snRNA-seq dataset from Wang *et al.*** The modules created in each pseudo-cell iteration is represented in different columns (from T_0_ to T_6_). In each iteration, modules of the corresponding iteration are represented as circles. The height of the circle represents the Z summary press. Under the null hypothesis of no module preservation, Z summary press<2 while a Z summary press >5 (or 10) indicates moderate (strong) module preservation (see dashed lines). The X-axis represents the size of the module. All iterations contain a gold module, which is a random module used as a reference of totally random module values. As observed, an average of 85.26% of the dopaminergic neurons modules are preserved in the Wang Qian *et al.* dataset in each pseudo-cell iteration.

**Supplementary Figure S6. Comparison of scCoExpNets with alternative pseudo-cell approaches using dopaminergic neurons expression data from the discovery dataset. (A)** Sparsity of the DN expression matrix after applying SEACells, hdWGCNA and scCoExpNets (the last one was applied while the number of pseudo-cell was above *m*). The height of the bar represents sparsity and over the bars is represented the number of pseudo-cell created. The three methods get reasonably similar ratios. **(B-H)** Pairwise comparisons between the gene modules detected with scCoExpNets and hdWGCNA approaches using Fisher’s exact test to test module overlaps. Only significant overlaps after p-value adjustment by FDR are colored. We observed that all modules within all GCNs created by scCoExpNets overlaps, at least partially, with one hdWGCNA gene module after p-value adjustment. * : *P*<0.01, **: *P*<0.05, ***: *P*<0.001

**Supplementary Table S1. Discovery cohort donors’ features.** Neuritic plaque abundance was scored using the CERAD score (0-3). Neurofibrillary tangles were scored using the Braak staging measure. CERAD: Consortium to Establish a Registry for Alzheimer's Disease; HC: healthy controls; PD: Parkinson’s disease; PMI: post-mortem interval; RIN: RNA integrity number.

| **Donor ID** | **Clinical diagnosis** | **Age of death** | **Sex** | **RIN** | **PMI (h)** | **Neuritic plaque abundance (CERAD score)** | **Neurofibrillary tangles abundance (Braak stage)** | **Lewy body presence in midbrain (1=YES)** | **Lewy body presence in limbic (1=YES)** | **Lewy body presence in neocortical (1=YES)** |
| --- | --- | --- | --- | --- | --- | --- | --- | --- | --- | --- |
| s.0096 | PD | 90 | female | 7.8 | 3.00 | 1 | 4 | 1 | 1 | 1 |
| s.0098 | PD | 99 | male | 7.0 | 3.00 | 1 | 4 | 1 | 1 | 1 |
| s.0102 | PD | 57 | female | 6.5 | 3.00 | 0 | 1 | 1 | 1 | 1 |
| s.0103 | PD | 68 | male | 6.4 | 61.00 | 1 | 4 | 1 | 1 | 1 |
| s.0104 | PD | 81 | male | 6.9 | 48.00 | 1 | 4 | 1 | 1 | 1 |
| s.0105 | PD | 78 | male | 6.5 | 12.00 | 0 | 1 | 1 | 1 | 0 |
| s.0107 | PD | 82 | male | 7.1 | 24.00 | 0 | 2 | 1 | 1 | 1 |
| s.0109 | PD | 98 | male | 7.0 | 20.50 | 0 | 3 | 1 | 1 | 1 |
| s.0110 | PD | 82 | male | 7.3 | 4.00 | 0 | 3 | 1 | 1 | 1 |
| s.0111 | PD | 80 | male | 7.5 | 46.00 | 1 | 2 | 1 | 1 | 1 |
| s.0116 | PD | 77 | male | 6.3 | 24.00 | 0 | 1 | 1 | 1 | 0 |
| s.0118 | PD | 85 | female | 7.7 | 40.00 | 1 | 4 | 1 | 1 | 1 |
| s.0119 | PD | 84 | male | 6.4 | 21.00 | 2 | 4 | 1 | 1 | 1 |
| s.0120 | PD | 85 | female | 7.5 | 24.00 | 0 | 3 | 1 | 1 | 1 |
| s.0128 | HC | 81 | female | 6.9 | 24.00 | - | - | - | - | - |
| s.0129 | HC | 86 | male | 6.7 | 13.00 | - | - | - | - | - |
| s.0130 | HC | 61 | male | 7.6 | 22.00 | - | - | - | - | - |
| s.0131 | HC | 65 | male | 7.0 | 22.00 | - | - | - | - | - |
| s.0142 | HC | 62 | male | 7.6 | 52.00 | - | - | - | - | - |
| s.0147 | HC | 30 | female | 8.0 | 12.00 | - | - | - | - | - |
| s.0151 | HC | 77 | female | 6.7 | 11.00 | - | - | - | - | - |
| s.0152 | HC | 93 | female | 7.7 | 8.00 | - | - | - | - | - |
| s.0154 | HC | 93 | male | 7.1 | 7.00 | - | - | - | - | - |
| s.0158 | HC | 89 | male | 7.7 | 18.75 | - | - | - | - | - |
| s.0159 | HC | 73 | female | 7.7 | 21.00 | - | - | - | - | - |
| s.0165 | HC | 91 | male | 7.2 | 17.75 | - | - | - | - | - |

**Supplementary Table S2. Curated list of SNpc DN markers obtained from scRNA-seq studies.**

DEGs: differentially expressed genes; GWAS: genome wide association studies; iDN: induced dopaminergic neurons; MB: midbrain; NS: not specified; SNpc: substantia nigra pars compacta; VTA: ventral tegmental area; WT: wild-type.

| **Reference** | **Specie** | **Brain region** | **Description** | **DEG** | **Genes** |
| --- | --- | --- | --- | --- | --- |
| Grimm *et al.* (2004) | Mouse | SNpc | DN SNpc vs VTA | UP | 10 |
|  |  |  |  | DOWN | 54 |
| Greene *et al.* (2005) | Mouse | SNpc | DN SNpc vs VTA | UP | 67 |
|  |  |  |  | DOWN | 57 |
| Chung *et al.* (2005) | Mouse | SNpc | DN SNpc vs VTA | UP | 57 |
|  |  |  |  | DOWN | 68 |
| Poulin *et al.* (2014) | Mouse | SNpc | DN SNpc vs VTA | UP | 19 |
| Bifsha *et al.* (2014) | Mouse | SNpc | DN SNpc vs VTA | UP | 20 |
|  |  |  |  | DOWN | 207 |
| Xia *et al.* (2016) | iPsc | - | DN PD vs control | DOWN | 13 |
|  |  |  |  | UP | 6 |
| LaManno *et al.* (2016) | Mouse | SNpc | DN SNpc vs VTA | UP | 17 |
|  |  |  |  | DOWN | 26 |
| Nichterwitz *et al.* (2016) | Human | SNpc | DN SNpc vs VTA | UP | 32 |
|  |  |  |  | DOWN | 60 |
| Hook  *et al.* (2018) | Mouse | SNpc | DN markers | DEG | 110 |
|  | ns | SNpc | DN literature | - | 49 |
| Skene *et al.* (2018) | Mouse | ns | DN markers | DEG | 33 |
|  |  | SNpc | DN markers | DEG | 25 |
| Kramer *et al.* (2018) | Mouse | SNpc | DN1 vs all cell types | UP | 27 |
|  |  |  | DN4 vs all cell types | UP | 25 |
| Agarwal *et al.* (2020) | Human | SNpc | DN vs all cell types | UP | 129 |
|  |  |  | DN vs neurons | UP | 77 |
| Fernandes *et al.* (2020) | iPsc | - | DN1 markers | UP | 172 |
|  |  |  | DN1 markers | DOWN | 126 |
|  |  |  | DN2 markers | UP | 163 |
|  |  |  | DN2 markers | DOWN | 152 |
|  |  |  | DN3 markers | UP | 174 |
|  |  |  | DN3 markers | DOWN | 116 |
| Bryois *et al.* (2020) | Human | - | PD genetic mutations | - | 66 |
|  | Human | SNpc | GTEx markers | DEG | 11 |
|  | Mouse | ns | Cholinergic markers | DEG | 42 |
| Zhong *et al.* (2021) | Mouse | MB | DN markers | UP | 32 |
|  |  |  | DN A53T vs WT | UP | 27 |
|  |  |  | DN A53T vs WT | DOWN | 30 |
| Aguila *et al.* (2021) | Human | SNpc | DN SNpc vs VTA | UP | 23 |
|  |  |  | DN SNpc vs VTA | DOWN | 51 |
|  |  |  | DN markers | DEG | 167 |

**Supplementary Table S3.** **Sepulveda cohort donors’ features.** PD: Parkinson’s disease; HC: healthy controls; RIN: RNA integrity number; PMI: post-mortem interval.

| **Donor ID** | **Clinical diagnosis** | **Age of death** | **Sex** | **RIN** | **PMI (h)** |
| --- | --- | --- | --- | --- | --- |
| s.0169 | PD | 71 | male | 7.2 | 21.5 |
| s.0170 | PD | 61 | male | 7.4 | 6.3 |
| s.0171 | HC | 70 | male | 7.8 | 11.8 |
| s.0172 | HC | 84 | male | 7.6 | 11.8 |
| s.0173 | HC | 88 | male | 8.5 | 9.3 |
| s.0176 | PD | 74 | male | 7.7 | 11.4 |
| s.0177 | PD | 79 | male | 7.9 | 12.3 |

**Supplementary Table S4. Cell types identified in the Oregon snRNA-seq dataset.**

GABA: Gamma-aminobutyric acid; GLU: glutamate.

| **Cell type** | **Genes detected**  **(CI 95%)** | **Sparsity (%)** | **Number of cells** | **Cell proportion (%)** |
| --- | --- | --- | --- | --- |
| Astrocytes | 1972 (1960-1984) | 79.76 | 23466 | 22.49 |
| Astrocytes/microglia | 2640 (2531-2748) | 66.63 | 256 | 0.25 |
| Connective tissue cells | 1909 (1804-2014) | 78.25 | 302 | 0.29 |
| Dopaminergic neurons | 1329 (1316-1343) | 80.79 | 6565 | 6.29 |
| Dopaminergic-like neurons | 1306 (1282-1330) | 84.08 | 2103 | 2.02 |
| Endothelial cells | 1773 (1741-1806) | 81.74 | 3080 | 2.95 |
| GABA neurons expressing GABA receptors | 1277 (1248-1306) | 80.95 | 1230 | 1.18 |
| GABA neurons expressing GLU receptors | 1562 (1482-1643) | 73.91 | 291 | 0.28 |
| GABA like neurons | 1623 (1573-1674) | 71.76 | 768 | 0.74 |
| GLU like neurons | 1571 (1526-1615) | 79.16 | 758 | 0.73 |
| Microglia | 1765 (1752-1778) | 75.26 | 15143 | 14.51 |
| Oligodendrocyte progenitor cells | 2063 (2041-2084) | 75.19 | 7029 | 6.74 |
| Oligodendrocytes | 1833 (1825-1841) | 78.94 | 42802 | 41.02 |
| T cells | 1701 (1649-1754) | 74.59 | 545 | 0.52 |

**Supplementary Table S5. Cell types identified in the Sepulveda snRNA-seq dataset.**

GABA: Gamma-aminobutyric acid.

| **Cell type** | **Genes detected**  **(CI 95%)** | **Sparsity (%)** | **Number of cells** | **Cell proportion (%)** |
| --- | --- | --- | --- | --- |
| Astrocytes | 2543 (2520-2566) | 73.44 | 4582 | 16.62 |
| Astrocytes/Oligodendrocytes | 1520 (1483-1558) | 85.25 | 741 | 2.69 |
| Connective tissue cells | 2052 (1925-2179) | 77.61 | 130 | 0.47 |
| Dopaminergic neurons | 1633 (1610-1655) | 80.12 | 567 | 2.06 |
| Dopaminergic-like neurons | 1689 (1619-1759) | 84.04 | 317 | 1.15 |
| Endothelial cells | 2341 (2240-2442) | 77.04 | 317 | 1.15 |
| GABA neurons | 2425 (2293-2558) | 64.58 | 68 | 0.25 |
| GABA-like neurons | 2196 (2103-2288) | 77.21 | 267 | 0.97 |
| Microglia | 2384 (2362-2406) | 71.52 | 3798 | 13.78 |
| Mural cells | 1729 (1659-1799) | 83.51 | 303 | 1.10 |
| Oligodendrocyte progenitor cells | 2799 (2762-2835) | 66.75 | 1674 | 6.07 |
| Oligodendrocytes | 2430 (2419-2441) | 69.42 | 14617 | 53.02 |
| T cells | 1882 (1819-1945) | 74.05 | 187 | 0.68 |

**Supplementary Table S6. T_0_ GO subgraphs maintained through all pseudo-cell iterations per cell type cluster.** See excel file.

**Supplementary Table S7. T_1_ new GO subgraphs not detected at T_0_ per cell type cluster.** See excel file.

**Supplementary Table S8. T_1_ GO subgraphs that expand or specialize T_0_ GO subgraphs per cell type cluster.** See excel file.

**Supplementary Table S9. Preservation of the M_1_ module from the DN GCN at T_0_ through pseudo-cell iterations in the Sepulveda dataset.** Under the null hypothesis of no module preservation, *Z*-summary score is below 2 while a *Z*-summary score greater than 5 (or 10) indicates moderate (strong) module preservation.

| **Pseudo-cell iteration** | **Module name** | **Module size** | **Z summary pres** |
| --- | --- | --- | --- |
| 0 | pink (M_1_) | 309 | 3.39 |
| 1 | purple | 316 | 1.57 |
| 2 | red | 380 | 2.64 |
| 3 | green | 417 | 2.07 |
| 4 | blue | 363 | 2.66 |
| 5 | blue | 364 | 2.85 |
| 6 | magenta | 305 | 2.29 |

**Supplementary Table S10. Functional enrichment of the M_1_ module from the DN GCN at T_0_.** See excel file.

**Supplementary Table S11. Fisher’s test results for the pairwise comparison of DN modules created with hdWGCNA and scCoExpNets methods.** See excel file.

**Supplementary Table S12. hdWGCNA GO subgraphs and its comparison with scCoExpNets GO subgraphs.** See excel file.
